# Supplementary figures and images for: PHEV infection: A promising model of betacoronavirus-associated neurological and olfactory dysfunction
Source: PLoS Pathog. 2022 Jun 27;18(6):e1010667. doi: 10.1371/journal.ppat.1010667 (PMC9282652; doi:10.1371/journal.ppat.1010667)

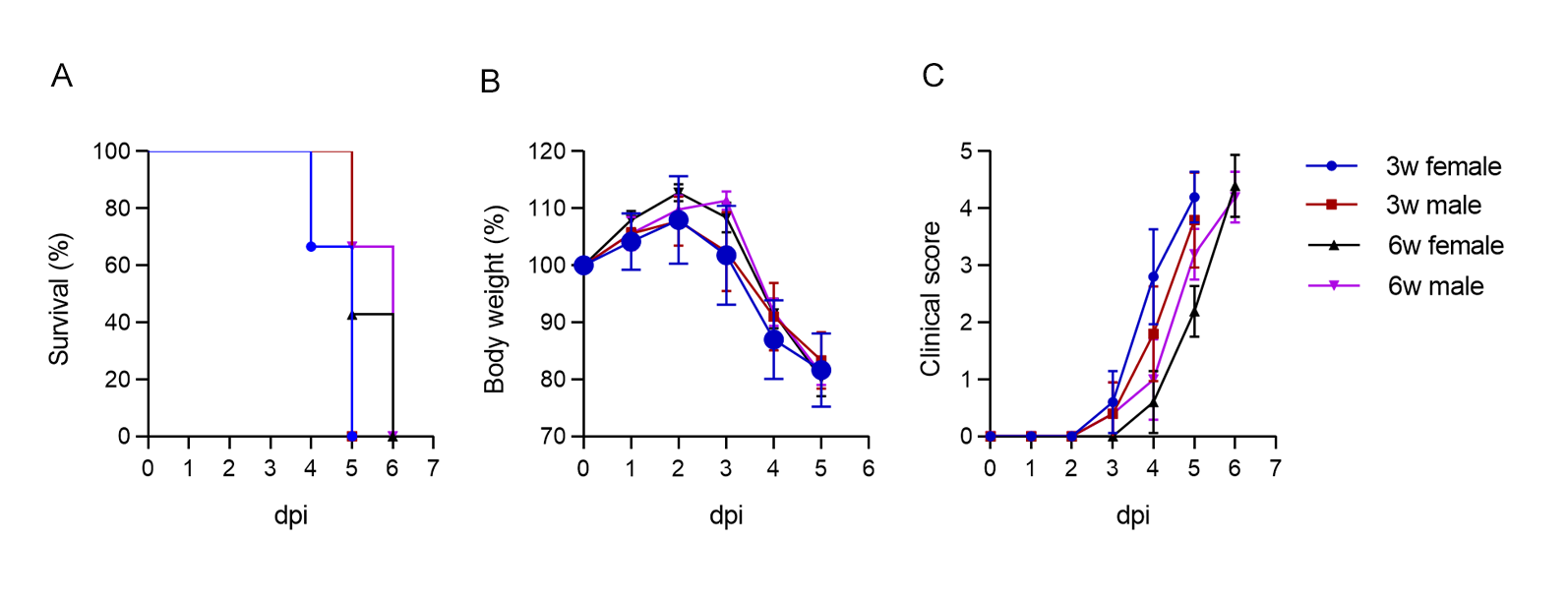

Supplement: S1 Fig — Male and female 3w and 6w BALB/c mice were intranasally inoculated with 103.96 TCID50 PHEV per mouse (n = 5 mice/age/sex). (A) Mortality of PHEV-infected female and male mice. (B) Percentage of initial body weight. (C) Clinical scores. P values were calculated by log-rank (Mantel–Cox) tests (survival) (A), Wilcoxon matched-pairs rank test (B), and one-way ANOVA (C). Data are presented as the means ± SD. (TIF) [file ppat.1010667.s001.tif]

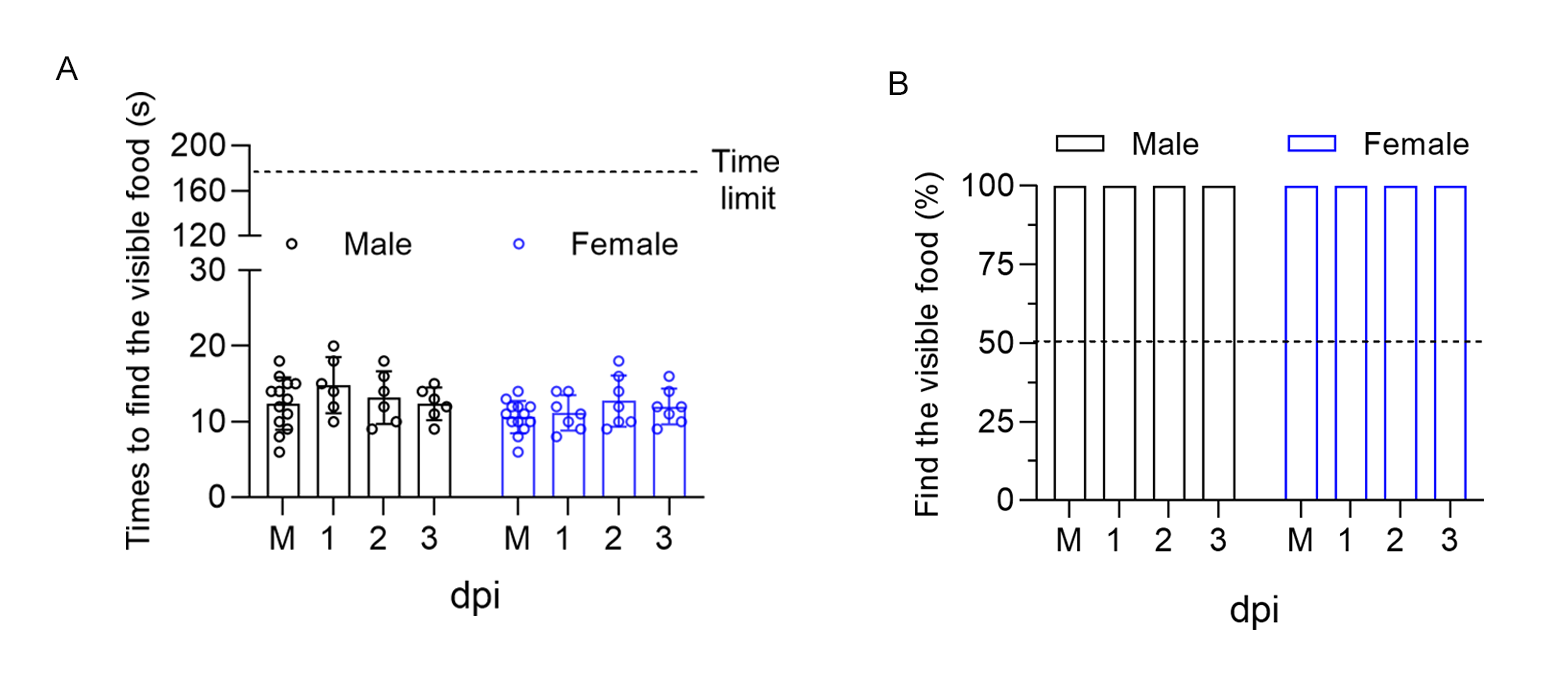

Supplement: S2 Fig — The 6w male and female BALB/c mice were intranasally inoculated with 103.96 TCID50 PHEV or mock-infected with PBS (M) before the visible food finding test. (A) Time spent by mock or infected mice (1–3 dpi) finding the visible food. The dashed line represents the time limit of 3 min. Each circle represents a mouse. (B) Percentage of mice that successfully found visible food within 3 min. For male mice (mock: n = 13, 1–3 dpi: n = 6), female mice (mock: n = 13, 1–3 dpi: n = 7). P values were calculated by one-way ANOVA (A). Data are presented as the means ± SD. (TIF) [file ppat.1010667.s002.tif]

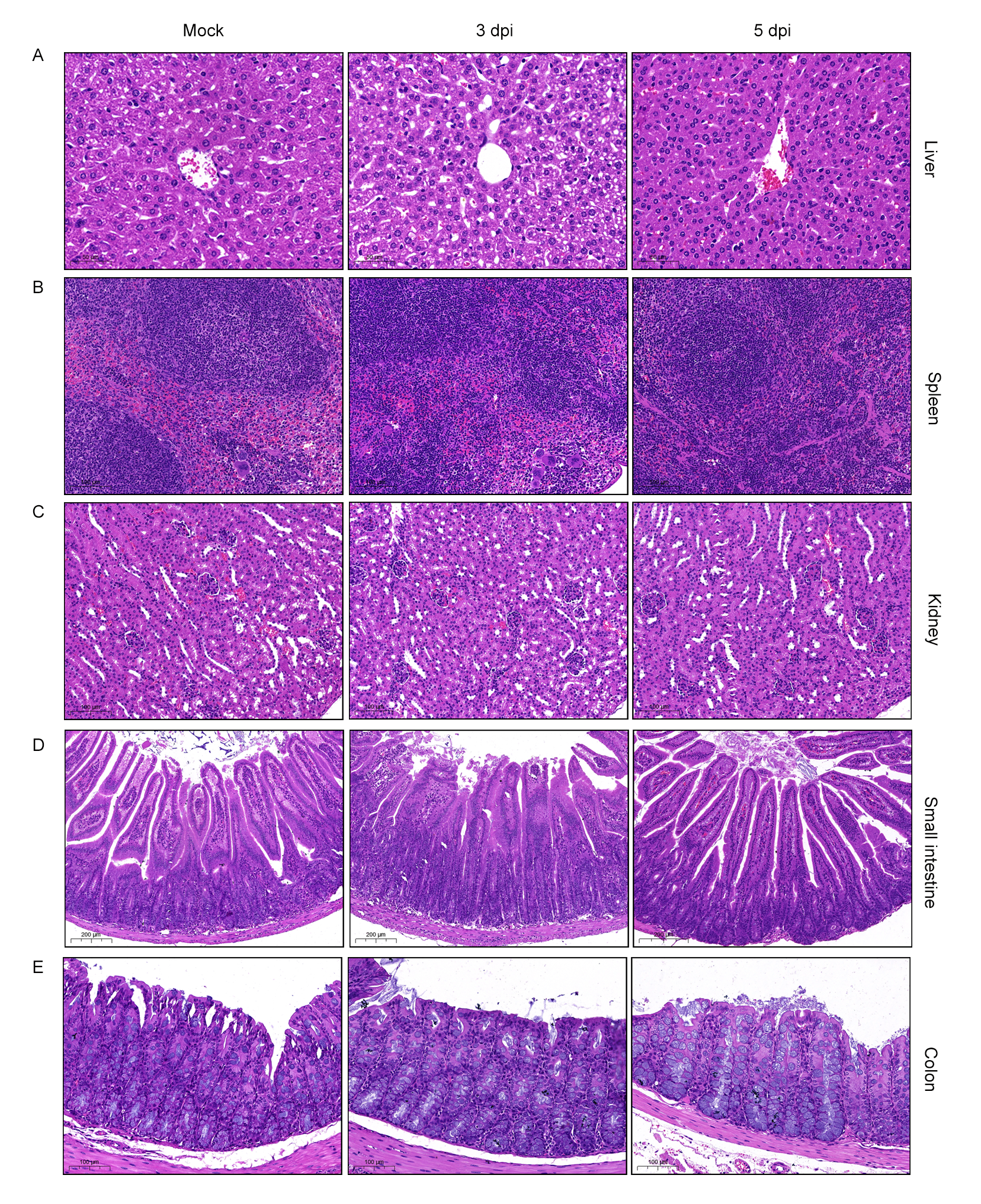

Supplement: S3 Fig — The 3w BALB/c mice were euthanized at 0, 3 and 5 dpi after 103.96 TCID50 PHEV inoculation, and tissues were collected for the histological examination. The liver (A), spleen (B), kidney (C), small intestine (D), and colon (E) were analyzed. There were no substantial histopathological changes in the organs of PHEV-infected mice. Scale bars, 50 μm (A), 100 μm (B, C and E), 200 μm (D), H&E staining. Two sections of each organ from 3 mice per group were analyzed. (TIF) [file ppat.1010667.s003.tif]

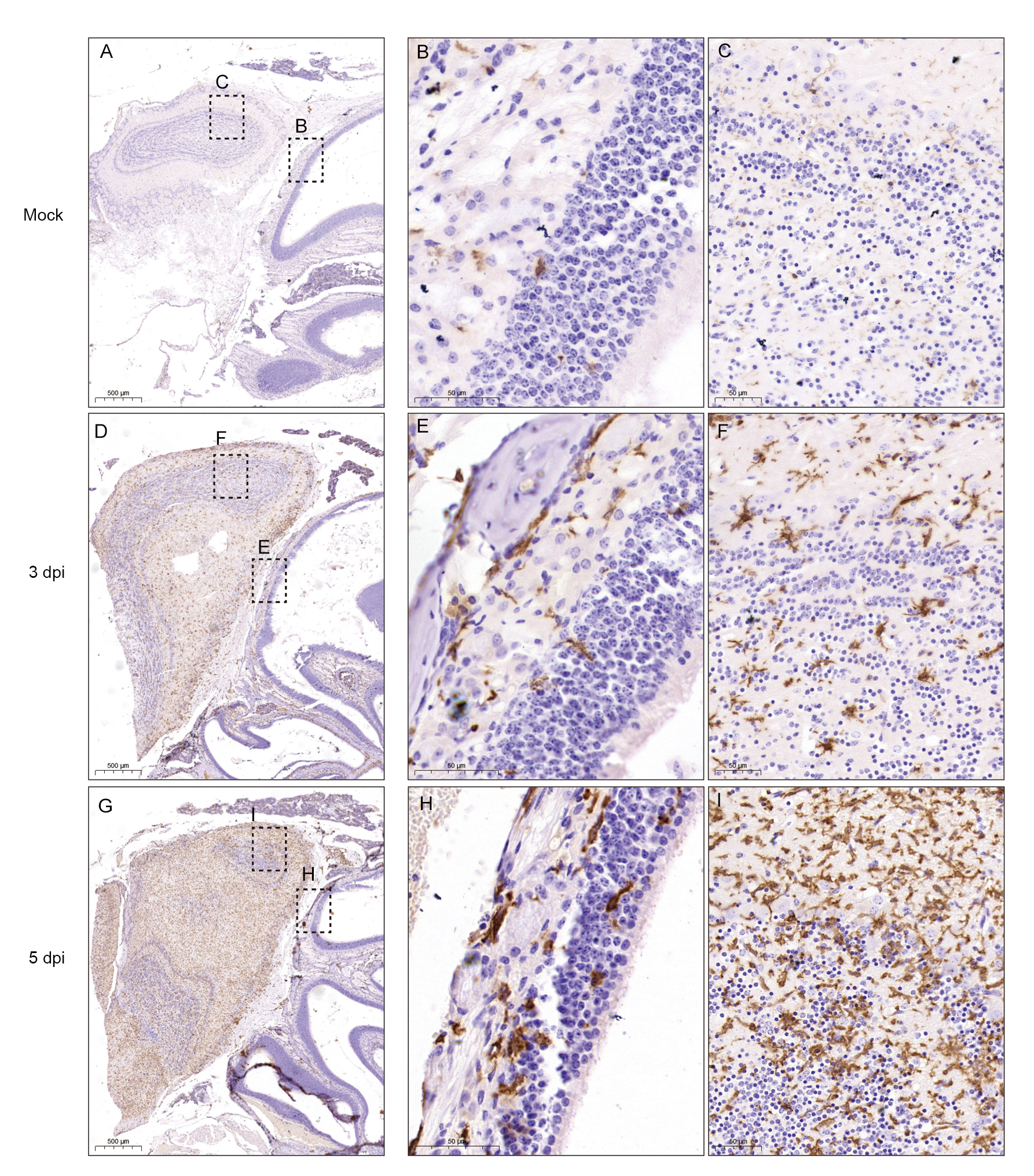

Supplement: S4 Fig — The 3w BALB/c mice were inoculated intranasally with 103.96 TCID50 PHEV. OB and nose tissues were collected at 0, 3 and 5 dpi for IHC with an antibody against IBA1. Compared with control tissues (A-C), incremental accumulation of IBA1-positive macrophages/microglia cells was observed in the OE and OB at 3 (D-F) and 5 (G-I) dpi. Scale bars, 500 μm (A, D, G), 50 μm (B, C, E, F, H, I). Two sections of each tissue from 3 mice per group were analyzed, and representative images are shown. (TIF) [file ppat.1010667.s004.tif]

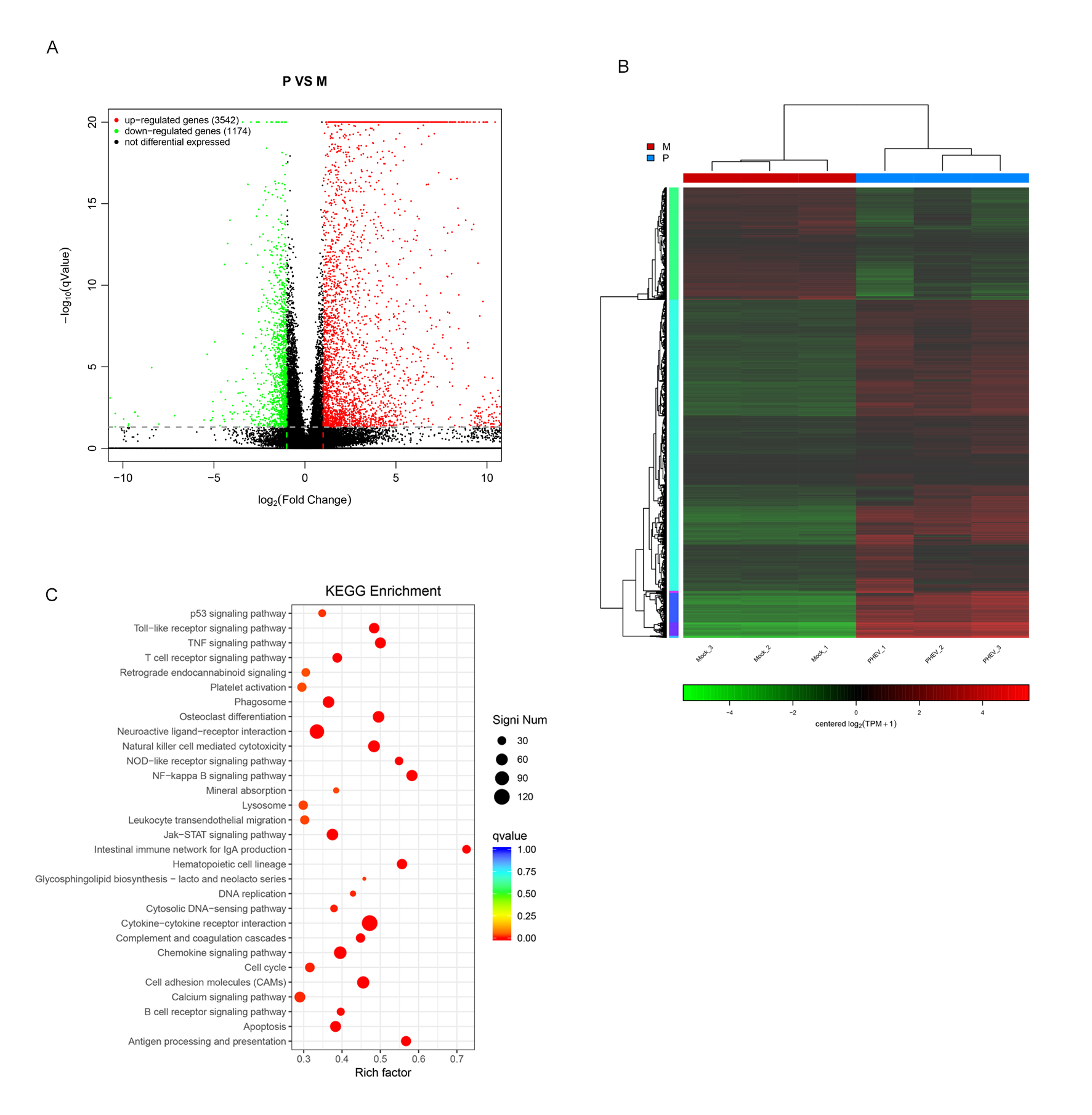

Supplement: S5 Fig — The 3w BALB/c mice were inoculated intranasally with 103.96 TCID50 PHEV and OB samples were collected at 5 dpi for RNA-seq. (A) Volcano plot. The horizontal axis represents the fold change in DEGs, and the vertical axis represents the Benjamini–Hochberg corrected p value on a logarithmic scale (-log10). Each dot represents a gene, where red dots represent up-regulated genes, green dots represent down-regulated genes, and black dots represent non-differentially expressed genes. b, Cluster heatmap of DEGs. Each row represents a gene, and each column represents a sample. The color represents the expression level of the gene, the red color represents a high expression level, and the green color represents a low expression level. c, Scatter plot of the KEGG pathway enrichment analysis. P, PHEV-infected sample; M, mock sample. (TIF) [file ppat.1010667.s005.tif]
