# Supplementary material for: PHEV infection: A promising model of betacoronavirus-associated neurological and olfactory dysfunction
Source: PLoS Pathog. 2022 Jun 27;18(6):e1010667. doi: 10.1371/journal.ppat.1010667 (PMC9282652; doi:10.1371/journal.ppat.1010667)
Supplement: S1 Table — (DOCX) [file ppat.1010667.s006.docx]

**S1 Table. Distribution of viral antigen in the brains of PHEV-infected mice.**

| **Brain region^§^** | **Viral antigen at the indicated time (dpi)^¶^** | | | | | **Brain region^§^** | **Viral antigen at the indicated time (dpi)^¶^** | | |
| --- | --- | --- | --- | --- | --- | --- | --- | --- | --- |
|  | **1** | | **3** | | **5** |  | **1** | **3** | **5** |
| **Olfactory bulb** |  | | | | | **Thalamus** |  | | |
| Olfactory nerve layer | - | + | | + | | Sensory-motor | - | + | +++ |
| Glomerular layer | - | + | | + | | Polymodal association | - | ++ | +++ |
| External plexiform layer | - | + | | ++ | |  |  |  |  |
| Mitral cell layer | - | ++ | | +++ | | **Hypothalamus** |  |  |  |
| Internal plexiform layer | - | + | | ++ | | Periventricular | - | + | ++ |
| Granule layer | - | ++ | | +++ | | Medial | - | ++ | +++ |
|  | | | | | | Lateral | - | + | +++ |
| **Cortex** |  | | | | | Median eminence | - | + | +++ |
| Frontal | - | +++ | | +++ | |  | | | |
| Somatomotor | - | +++ | | +++ | | **Midbrain** |  | | |
| Somatosensory | - | + | | +++ | | Sensory | - | + | +++ |
| Gustatory | - | ++ | | +++ | | Motor | - | ++ | +++ |
| Visceral | - | ++ | | +++ | | Behavioral state | - | ++ | +++ |
| Visual | - | +++ | | +++ | |  | | | |
| Cingulate | - | ++ | | +++ | | **Hindbrain** |  | | |
| Prelimbic | - | +++ | | +++ | | pons | - | ++ | +++ |
| Infralimbic | - | +++ | | +++ | | medulla | - | ++ | +++ |
| Orbital | - | +++ | | +++ | |  | | | |
| Agranular insular | - | ++ | | +++ | | **Cerebellum** |  | | |
| Retrosplenial | - | + | | ++ | | Purkinje layer | - | - | + |
| Parietal | - | ++ | | +++ | | Granular layer | - | - | ++ |
| Temporal | - | + | | ++ | | Molecular layer | - | - | ++ |
| Ectorhinal | - | + | | ++ | |  | | | |
| Piriform | - | +++ | | +++ | | **Ventricular system** |  | | |
|  | | | | | | Lateral ventricle | - | - | - |
| **Hippocampus** |  | | | | | Third ventricle | - | - | - |
| CA1-4 | - | ++ | | +++ | | Cerebral aqueduct | - | - | - |
| Dentate gyrus | - | + | | +++ | | Fourth ventricle | - | - | - |

**^§^** Brain regions are referenced to ALLEN Brain Atlas: <http://mouse.brain-map.org/static/atlas>.

**^¶^** -, no antigen detected; +, 1 to 5 positive cells/region; ++, 6 to 20 positive cells/region; and +++, >20 positive cells/region.
