# Supplementary material for: PHEV infection: A promising model of betacoronavirus-associated neurological and olfactory dysfunction
Source: PLoS Pathog. 2022 Jun 27;18(6):e1010667. doi: 10.1371/journal.ppat.1010667 (PMC9282652; doi:10.1371/journal.ppat.1010667)
Supplement: S2 Table — (DOCX) [file ppat.1010667.s007.docx]

**S2 Table**. **Comparison of anosmia and brain infection among the PHEV-infected mouse model, SARS-CoV-2-infected hamster model, SARS-CoV-2-infected humanized ACE2 mouse model and deceased COVID-19 patients.**

| **Model** | **RE** | **OE** | **OB** |
| --- | --- | --- | --- |
| PHEV /Mouse | Infection, inflammatory response, target cells unidentified | Infection, inflammatory response, mainly target OSNs | Infection, inflammatory response, target neurons |
| SARS-CoV-2  /Mouse[1-3] | Infection in K18-hACE2 mice, unidentified in hACE2 knockin mice | Infection, inflammatory response, target OSNs, bowman’s gland cells, and sustentacular cells | Infection in K18-hACE2 mice, no infection in hACE2 knockin mice |
| SARS-CoV-2 /Hamster**[**4-7] | Unidentified | Infection, inflammatory response, loss of ciliation, target sustentacular cells, OSNs, and immune cells | Infection, inflammatory response, target immune cells and unidentified cells |
| COVID-19 patients[8] | Infection, target ciliated cells | Infection, target sustentacular cells | No infection |

Abbreviations: RE, respiratory epithelium; OE, olfactory epithelium; OB, olfactory bulb; OSN, olfactory sensory neuron; Iba 1, ionized calcium binding adapter molecule 1.

**Reference:**

1. Zheng J, Wong L-YR, Li K, Verma AK, Ortiz ME, Wohlford-Lenane C, et al. COVID-19 treatments and pathogenesis including anosmia in K18-hACE2 mice. Nature. 2020;589(7843):603-7. doi: 10.1038/s41586-020-2943-z.

2. Ye Q, Zhou J, He Q, Li RT, Yang G, Zhang Y, et al. SARS-CoV-2 infection in the mouse olfactory system. Cell Discov. 2021;7(1):49. Epub 20210706. doi: 10.1038/s41421-021-00290-1. PubMed PMID: 34230457; PubMed Central PMCID: PMCPMC8260584.

3. Sun SH, Chen Q, Gu HJ, Yang G, Wang YX, Huang XY, et al. A Mouse Model of SARS-CoV-2 Infection and Pathogenesis. Cell host & microbe. 2020;28(1):124-33 e4. Epub 20200527. doi: 10.1016/j.chom.2020.05.020. PubMed PMID: 32485164; PubMed Central PMCID: PMCPMC7250783.

4. de Melo GD, Lazarini F, Levallois S, Hautefort C, Michel V, Larrous F, et al. COVID-19-related anosmia is associated with viral persistence and inflammation in human olfactory epithelium and brain infection in hamsters. Science translational medicine. 2021;13(596). Epub 2021/05/05. doi: 10.1126/scitranslmed.abf8396. PubMed PMID: 33941622; PubMed Central PMCID: PMCPMC8158965.

5. Bryche B, St Albin A, Murri S, Lacote S, Pulido C, Ar Gouilh M, et al. Massive transient damage of the olfactory epithelium associated with infection of sustentacular cells by SARS-CoV-2 in golden Syrian hamsters. Brain Behav Immun. 2020;89:579-86. Epub 2020/07/07. doi: 10.1016/j.bbi.2020.06.032. PubMed PMID: 32629042; PubMed Central PMCID: PMCPMC7332942.

6. Zhang AJ, Lee AC-Y, Chu H, Chan JF-W, Fan Z, Li C, et al. Severe Acute Respiratory Syndrome Coronavirus 2 Infects and Damages the Mature and Immature Olfactory Sensory Neurons of Hamsters. Clinical Infectious Diseases. 2020. doi: 10.1093/cid/ciaa995.

7. Reyna RA, Kishimoto-Urata M, Urata S, Makishima T, Paessler S, Maruyama J. Recovery of anosmia in hamsters infected with SARS-CoV-2 is correlated with repair of the olfactory epithelium. Scientific reports. 2022;12(1):628. Epub 20220112. doi: 10.1038/s41598-021-04622-9. PubMed PMID: 35022504; PubMed Central PMCID: PMCPMC8755745.

8. Khan M, Yoo SJ, Clijsters M, Backaert W, Vanstapel A, Speleman K, et al. Visualizing in deceased COVID-19 patients how SARS-CoV-2 attacks the respiratory and olfactory mucosae but spares the olfactory bulb. Cell. 2021;184(24):5932-49 e15. Epub 20211103. doi: 10.1016/j.cell.2021.10.027. PubMed PMID: 34798069; PubMed Central PMCID: PMCPMC8564600.
