# Supplementary material for: PHEV infection: A promising model of betacoronavirus-associated neurological and olfactory dysfunction
Source: PLoS Pathog. 2022 Jun 27;18(6):e1010667. doi: 10.1371/journal.ppat.1010667 (PMC9282652; doi:10.1371/journal.ppat.1010667)
Supplement: Attachment — Submitted filename: Response to reviewers-20220510lz.docx [file ppat.1010667.s008.docx]

**Response Letter**

Dear Editors,

Thank you very much for your kind letter. We have carefully revised the manuscript in accordance with the reviewers’ comments. All concerns and questions have been addressed point-by-point below.

Kind regards,

Dr. Wenqi He

Professor of College of Veterinary Medicine, Jilin University

On behalf of all authors.

**Responses to Part I - Summary**

**Reviewer #1:**

In this manuscript by Shi et al, authors utilize the BALB/c mouse model to investigate the infectivity and tropism of a porcine betacoronavirus (PHEV) in neurological and olfactory-associated tissues. Authors conduct several behavioral studies to measure changes in CNS-related functions in infected mice, and extensively employ immunohistochemistry-based assessments to identify specific tissue structures post-inoculation that likely contribute to these in vivo readouts. They identify specific nerves targeted by PHEV infection and link viral replication at these sites with pathogenic responses. The study appears generally well-controlled, though there are places in the text that make it difficult to follow what exactly is being shown. The manuscript is generally well-referenced and logically organized, with a few exceptions. However, the authors heavily interpret their results in the context of SARS-CoV-2 infection, at the expense of focusing on what their findings mean in the context of PHEV infection. Overall there are several areas throughout the manuscript that warrant improvement.

**Response:** Thank you for your insightful comments. We have considered your advice and carefully revised the manuscript according to each point. We hope the revised manuscript has been substantially improved.

**Reviewer #2:**

In the manuscript by Shi et al the authors investigate the neuroinvasion routes for the betacoronavirus called Porcine hemagglutingating encephalomyelitis virus (PHEV) using a BALB/c murine system. Their work provides mechanistic understanding regarding PHEV neuroinvasion and neurotropism. The main findings are that the virus can invade the CNS via the olfactory epithelium/olfactory nerve, in addition to via the trigeminal nerve. The findings are convincing and the studies are well executed. This work adds to our understanding of PHEV and its ability to invade the CNS. However, the authors do not discuss how this work relates to PHEV pathogenesis, despite this virus being an important agricultural pathogen and a major source of economic loss on many farms (Mora-Diaz, et al; Front Vet Sci. 2019 doi: 10.3389/fvets.2019.00053). Rather, these data are postulated to represent a low containment model to study SARS-CoV-2 neuropathogenesis. Here the authors also fall short because they do not adequately related their findings to relevant SARS-CoV-2 models, most importantly the hamster models that have provided insight into anosmia and brain infection that is observed in humans. The manuscript also needs a clear discussion pertaining to the limitations of the surrogate PHEV model as it relates to SARS-CoV-2.

**Response:** We are very grateful for the comments from you. Based on your advice, we have carefully revised the manuscript and paid more attention to the link between our work and PHEV pathogenesis. We have discussed the relevance of our findings to SARS-CoV-2 models (Lines 281-288, 310-317, 363-370), and provided a table comparing anosmia and brain infection among the PHEV-infected mouse model, SARS-CoV-2-infected hamster model, SARS-CoV-2-infected humanized ACE2 mouse model, and deceased COVID-19 patients (S2 Table). In addition, we have added a discussion of the limitations to the Discussion section in the revised manuscript (Lines 396-402).

**Reviewer #3:**

The manuscript“PHEV infection: a promising model of betacoronavirus-associated neurological and olfactory disfunction” by Shi et al. is describing at mouse model to study the neuroinvasion and neurotropism about PHEV. The authors give a detailed description about pathogenicity and time course of infection and give a great overview of the mechanism of CNS infection of PHEV. Although this manuscript presents a comprehensive set of new data, the description of experiments and the interpretation of results is making it hard for the reader to follow the thoughts of the authors.

**Response:** We are grateful to receive these reviews from you, and we have addressed the issues related to the experimental description and interpretation of the results in the revised manuscript. We hope that our revision provides clear descriptions of the methods and results.

**Responses to Part II – Major Issues: Key Experiments Required for Acceptance**

**Reviewer #1:**

1. The majority of the introduction and discussion sections pertain to SARS-CoV-2 infection, not PHEV infection. It is understandable that this study with PHEV was undertaken to provide contextual information to SARS-CoV-2 infection, but without side-by-side data showing that SARS-CoV-2 and PHEV infection target similar tissues or elicit comparable responses, it’s difficult to use the PHEV model established here as a surrogate virus to directly inform SARS-CoV-2/COVID-19 studies. What receptor(s) does PHEV employ to infect CNS-related cells, what is their relative distribution on the specific CNS-associated tissues employed in this study, and are SARS-CoV-2 receptors present on the same tissues in permissive species? This is all critical information that appears to be missing or underemphasized within the text. While SARS-CoV-2 cannot infect BALB/c mice (limiting side-by-side assessments of both viruses in the same model), providing some scope of data regarding which mammalian models would be appropriate for employing any subsequent results obtained in the PHEV mouse model discussed here seems appropriate; without this context and additional information, the authors should temper their applicability of PHEV results to SARS-CoV-2 infection studies.

**Response:** Thank you for your suggestions.

(1) We have added more information about PHEV infection and pathogenesis in the Introduction Section (Lines 71-108) and discussed the relevance of our findings to SARS-CoV-2 models in the Discussion Section (Lines 281-288, 310-317, 363-370) of the revised manuscript. And also, we have provided a table comparing anosmia and brain infection among the PHEV-infected mouse model, SARS-CoV-2 infected hamster model, SARS-CoV-2 infected humanized ACE2 mouse model, and deceased COVID-19 patients (S2 Table). The corresponding descriptions have been added to the Discussion section of the revised manuscript (Lines 363-372).

(2) Research on the entry receptors for PHEV is ongoing. In 1990, B. Schultze et al. reported that PHEV attaches to N-acetyl-9-O-acetylneuraminic acid-containing receptors on erythrocytes (PMID: 2385959). Later, NCAM, which is expressed on the surface of neurons, glia, skeletal muscle, and natural killer cells, was shown to interact with PHEV and promote its entry into nerve cells (PMID: 20863409; PMID: 25925196). In addition, cell-surface glycans, i.e., sialic acid (SA) and heparan sulfate (HS), act as attachment factors for PHEV in nerve cells (PMID: 34972029) (Lines 90-95). SARS-CoV-2 uses ACE2 as a receptor for entry and the serine protease TMPRSS2 for S protein priming (PMID: 32015507; PMID: 32142651). Other than ACE2, neuropilin-1 (NRP1) also represents a host factor mediating SARS-CoV-2 infection (PMID: 33082294). NRP1 is abundantly expressed in nearly every cell type in the nasal passages, olfactory neurons in the olfactory bulb and olfactory tracts, and neurons in the CNS, where PHEV infections mainly occur (PMID: 33395426 and 33000221). Further research is needed to clarify whether NRP1 serves as a host factor for PHEV infection. We have added this information to the revised manuscript (Lines 314-317).

2. The methods section is lacking the humane endpoints employed in this study (were mice euthanized once they reached specific endpoint cutoffs)? The survival curves presented are difficult to interpret without understanding which specific criteria (weight loss, lethargy, specific neurological symptoms, etc) necessitated this euthanasia. It is also unclear as currently written if the mice that succumbed to infection in Fig 4A (with delays in death relative to Figure 1B) were due to the same manifestation of endpoint criteria or if the prolonged course of infection resulted in additional sequalae to develop. The method of humane euthanasia also needs to be disclosed (chemical, physical, etc).

**Response:** Thank you for your suggestions. We have modified the statement in the Introduction and Discussion sections of the revised manuscript.

(1) Based on your comments, we have added an ethics statement (Lines 413-417) and the method of humane endpoints (Lines 454-459 and Table 1) to the Materials and methods section of the revised manuscript. Humane endpoints were established based on the IACUC approved clinical scoring system (PMID: 35336942). Mice were considered moribund and humanely euthanized when the cumulative clinical score of 4 or weight loss greater than or equal to 20% was observed. Mice were euthanized when they reached humane endpoints. Briefly, mice were exposed to 5% isoflurane for 5 min in a plexiglass chamber before being decapitated when fully sedated, as measured by the absence of an active paw reflex (PMID: 30983972; PMID: 34480936).

(2) We apologize that the description of endpoint criteria in Fig 4A (revised Fig 6A) was not convincing and clear. We used the same criteria for humane endpoints in Fig 4A (revised Fig 6A) and Fig 1B, and corresponding descriptions have also been added to the Materials and methods section (Lines 501-502). The mice that succumbed to infection in Fig 4A with delays in death were due to the chemical destruction of olfactory nerve endings in the OE by ZnSO_4_ or Triton X-100 exposure. No obvious additional sequalae were observed in the prolonged course of infection.

3. As described in the text, Figure 1 purports to show differences between mock-infected and PHEV-infected mice, but many of the display images (1D-O) only appear to show breakdowns between male and female mice, and not infected vs mock. Figure legend for Fig 1 also states that bars represent mean + SD, but SD does not appear to be included in panels E-F, making it difficult for the reader to understand if the bars represent a different parameter or not.

**Response:** In the original study, we compared the difference in performance between mock-infected and PHEV-infected mice, as well as between female and male mice. Unfortunately, the original histogram did not accurately display the corresponding data. Based on your advice, we have redrawn the histograms and revised the description of the experimental results in more detail to match the figures. Please see the revised Fig 2 and Lines 135-155 in the revised manuscript. In addition, error bars have been added to the original Fig.1 E-F (currently revised Fig 2B-C).

**Reviewer #2:**

1. While the authors cite the hamster model papers that examined SARS-CoV-2 in the olfactory epithelium and brain infection (de Melo at al), there is virtually no discussion about the hamster SARS-CoV-2 model or these cited studies. The hamster system is extensively used for SARS-COV-2 research, including infection in the olfactory cavity. I think this should be at least a paragraph in the discussion addressing the hamster models (and other models) and a comparison to their results. Ideally, the authors should consider a table showing how the PHEV BALB/c model and the hamster models relate to what has been identified in humans vis-a-vis anosmia and infection of the olfactory bulb.

**Response:** We agree. According to your suggestion, we have discussed this issue in the Discussion section in the revised manuscript (Lines 363-375). Moreover, a table comparing anosmia and brain infection among the PHEV-infected model, SARS-CoV-2-infected models, and deceased COVID-19 patients has been added in the revised manuscript (S2 Table).

2. There appears to be no lung involvement in the PHEV/BALB/c system. Some evidence suggests that SARS-COV-2 can usurp the vagus nerve subsequent to lung infection to invade the CNS (Bulfamante, G et al J. Neurol; 10.1007/s00415-021-106 04-8). Does a lack of respiratory involvement impair the in the PHEV system as a surrogate for SARS-CoV-2 neuroinvasion? This should be considered in a limitations paragraph along with other pros and cons of the PHEV surrogate model.

**Response:** Thank you for your comments. In fact, no lung involvement was observed in the PHEV-infected BALB/c mice. The main objective of our study was to investigate PHEV neuroinvasion in mice and its potential use as a promising model to clarify SARS-COV-2-associated neurological and olfactory and taste dysfunction. Three-week-old mice were treated with PHEV intranasally, and viral genomic loads in the lung were measured to address your concern. Brain samples served as a positive control. In the brain, viral N genomic RNA was initially detected at 3 dpi and up to 5 dpi when mice died; however, no viral RNA was detected in the lungs throughout the infection (Fig. R1a-b). Compared with the control group, no obvious histopathological changes or Iba1 positive inflammatory cell infiltration was observed in the lungs after PHEV infection (Fig. R1c-n). Similarly, PHEV did not alter cytokine and chemokine abundance in the lung compared with brain tissues (Fig. R2). Thus, we concluded that PHEV-infected mice had minimal to no pathological changes in their lungs, consistent with their undetectable viral load. As suggested, we have added a limitations paragraph to the revised manuscript (Lines 330-339).

**Fig R1. PHEV infection induced minimal to no histopathological changes in the lungs of BALB/c mice.** Three-week-old BALB/c mice were inoculated intranasally with 10^3.96^ TCID_50_ PHEV and sacrificed at 1-5 dpi. **(a-b)** Viral N genomic RNA loads in brain and lung samples were detected daily at 1 to 5 dpi by qRT-PCR and RT-PCR. (**c-h**) Lungs from control and infected mice were stained with H&E to show histopathological changes. (**i-n**) Lungs from control and infected mice were immunostained with an Iba1 antibody to show inflammatory cell infiltration.

**Fig R2. Levels of inflammatory cytokines and chemokines in the lungs and brains of PHEV-infected mice.** Three-week old BALB/c mice were inoculated intranasally with 10^3.96^ TCID_50_ PHEV and sacrificed at 1, 3, and 5 dpi (3 mice per time point). The mRNA levels of cytokines and chemokines in the lungs and brains were determined by qRT-PCR. Brain samples served as a positive control. The Y axis represents the fold change in the infected mice compared with the control mice. The mRNA levels of target genes were normalized to the level of GAPDH and the data are presented as the means ± SD. *P* values were calculated by ANOVA, two-tailed Student’s t test. **p* < 0.05; ***p* < 0.01; ****p* < 0.001.

3. The introduction and discussion should clearly and conspicuously address the agricultural disease caused by PHEV, including the fact that it is an important pathogen. How the author’s data relates to what is known about this disease should also be added to the discussion in its own paragraph. The authors may want to consider reformatting the manuscript as a PHEV pathogenesis paper, then mention its potential use as a surrogate SARS-COV-2 system in the dissuasion.

**Response:** We agree. Based on your and the other reviewers’ advice, we have rephrased the Introduction and Discussion sections in the revised manuscript (Lines 59-117 and Lines 277-409).

4. The discussion mentions the potential involvement of ACE2 binding as being important for loss of taste, etc. However, I don’t believe that PHEV binds ACE2, rather it binds sialic acid and potentially the Neural Cell Adhesion Molecule. If binding to ACE2 were critical for SARS-CoV-2 neuroinvasion, but PHEV didn’t use this receptor, it would make this a poor model to mimic COVID-19. The authors need to explicitly state the receptor that PHEV uses and then modify the discussion as to how this may be a limitation to this model.

**Response:** Thank you for your suggestion. We have modified the statement in the revised manuscript.

(1) According to you suggestion, we have added the information of receptor usage of PHEV (Lines 90-93).

(2) The exact mechanisms of taste dysfunction caused by coronaviruses have not yet been clarified. The potential involvement of ACE2 binding may relate to taste loss, however there is no experimental data to support this hypothesis. PHEV does not appear to use ACE2 as a cell entry receptor, so the mechanisms of PHEV-induced ageusia remains to be elucidated in the future (Lines 381-382).

(3) In this study, we proposed that the pathogenesis of PHEV-associated neurological complications and olfactory and taste disorders may provide references for other coronaviruses, albeit with limitations. The difference in the host cell receptors used between PHEV and other human coronaviruses precludes studies of viral entry or drugs that act on this stage of the replication cycle. We have added this limitation in the discussion section in the revised manuscript (Lines 398-402).

5. It isn’t clear why the authors used 3 and 6 week old mice initially, then changed over to just 3 week old mice for subsequent studies. 6 week old mice would be preferred as these are adult mice. Is there a reason to only have used 3 week old mice?

**Response****:** We regret that this information was not clearly explained in our original description. Actually, 6-week-old BALB/c mice were only used in behavioral experiments and 3-week-old BALB/c mice were used for the rest of the neuroinvasion test (revised Fig.1A and Lines 405-412).

In this study, 3- and 6-week-old mice were used to simulate the infection of young and sexually mature hosts respectively. Because PHEV mainly infects young piglets within 3-week-old in clinical practice (PMID: 30873421), and 3-week-old mice were used in most studies of PHEV (PMID: 31176401; 34643429 and 35082791), so we used 3-week-old mice for the neuroinvasion test. However, in behavioral experiments, previous studies recommend using mice at least six weeks of age because juveniles (3-week) may respond differently to adult (6-week) mice (PMID: 29988104; 19575474 and 33166988).

**Reviewer #3:**

1. I am missing a consistency of methodological workup of samples in terms of titration and PCR. For some samples, titrations are performed to quantify infectious virus, for some not. Either explain your workflow or keep it consistent throughout all experiments

**Response:** Thank you for your suggestion. As shown in the revised Fig 3A-B, we found that the value change tendency of titration and qRT-PCR have no significant difference, thus we only used qRT-PCR to analyze viral antigen distribution in the CNS in subsequent studies. We would like to supplement the viral titer testing if you deem it necessary. However, our lab is now closed due to the impact of COVID-19. Please give us an extra month to complete this work.

2. It is very hard to follow which animals (gender, age) and how many (n=?) were used for the different experiments. It seems as if a lot of different sub-experiments were performed with partially different conditions. It would be ideal for reader to follow the workflow and thoughts of the authors if a graphical experimental setup or a table would give an overview of those aspects.

**Response:** Thank you for your suggestion. In order to easy follow the workflow, a graphical experimental setup (revised Fig.1A) and a description of the study design were included in the Materials and methods section (Lines 405-412). In addition, we have clarified this information in all figure legends in the revised manuscript.

**Responses to Part III – Minor Issues: Editorial and Data Presentation Modifications**

**Reviewer #1:**

1. Abstract and author summary require editing for English prose (furthermore, use of “biosafety” and “biosafe” in the abstract do not make logical sense as current written). There are also some passages within the main text where phrasing is too colloquial (e.g. line 100, “attractive”, line 152 “suffering mice”).

**Response:** We agree. We have invited senior English experts in the field to edit the manuscript and carefully revised the full text. We hope the revised manuscript has been substantially improved.

2. anosmia and ageusia should be better defined in the author summary as this is intended for a more general audience.

**Response:** Agree. We have modified the statement in the revised manuscript (Lines 43-44).

3. lines 127-8, what additional “neurological complications” are being alluded to outside of anosmia and ageusia?

**Response:** The neurological complications caused by PHEV included ruffled fur, hunchback posture, tremors, and ataxic gait. This information is presented in Lines 129-130 in the revised manuscript.

4. Authors should be commended for employing both male and female mice of two ages in this study, however, it is unclear why authors elected to use the 3 week vs the 6 week old mice in different studies. Was there a research purposes for choosing one age over another in selected studies (e.g. studies shown in Figure 3, line 147) or were findings interchangeable? If there was a reason, the authors should better disclose this for clarity. There are also places the age of mice employed is not clearly stated (e.g. Figure 5 legend)

**Response****:** Clinically, PHEV mainly affects suckling piglets under 4 weeks of age. Thus, we selected young mice at 3 and 6 weeks of age to mimic clinical infection. For the behavioral experiments (revised Fig.2), we selected 6-week-old mice for testing because these mice had fully developed organs and reached sexual maturity (PMID: 29988104; PMID: 33166988). Compared with 3-week-old mice, the behavioral evaluation of 6-week-old mice is more stable and reliable. For remaining viral neuroinvasion test, we used immature 3-week-old mice because of the higher efficiency of PHEV invasion in younger mice. Based on your comments, we have added additional explanations to the revised manuscript. Please see Lines 135-136 and Lines 164-165 in the revised manuscript. In addition, we have carefully revised all of the figure legends and clearly stated the age of mice employed.

5. Figure 4, it is unclear if the data presented is from ZnSO_4_-treated mice or not (e.g. Figure 4B, the text implies that the treated mice are being shown, but this is not specified in the legend text, and then panels 4C-F seem to imply that what is being shown is from non-treated mice). If ZnSO_4_-treated mice are being shown, showing both treated- and untreated- mice here would seem appropriate so that the reader can better appreciate differences that are specifically attributed to the destroyed olfactory nerve endings induced by ZnSO_4_.

**Response:** We agree. In order to easy follow for readers, we have updated the original Fig 4B (i.e., revised Fig 6B-D) and revised the text in the revised manuscript (Lines 208-214 and 957-965). In the original Fig 4C-F (i.e., Revised Fig 7 and 8), these data are used to show the roles of trigeminal nerve and neural axon, and mice in these experiments were not treated with ZnSO_4_. We have re-edited the figure legends in the revised manuscript (Lines 966-978).

6. Figure 5D, what is fold induction relative to? Uninfected mice? The methods, figure legend, or text do not specify this. It would be beneficial if baseline quantities of each analyte examined were disclosed in the legend or methods so that the reader could better understand the relative expression (and magnitude of change when appropriate) for each.

**Response:** Agree. Data were normalized to GAPDH and presented as fold changes in expression relative to mock-infected mice (Line 995-997). We have added this statement to the figure legend in the revised manuscript (revised Fig 10C).

**Reviewer #2**:

1. line 26, “a biosafety betaconronavirus” does not make sense. This should be reworded. Maybe “non-human pathogenic..”?

**Response:** Yes. We have deleted this statement in the revised manuscript.

2. Line 41-43. “...utilized as a prototype to recapitulate neurological and olfactory dysfunction in a subset of COVID-19 involvement under...” should be reworded. Perhaps “....olfactory dysfunction observed in human SARS-COV-2 infections?

**Response:** Yes. We have reworded the statement in Lines 47-49 of the revised manuscript.

3. Line 106. The strain of virus used should be added to be consistent (CC14?).

**Response:** Yes. PHEV strain CC14 is now added (Line 122-123).

4. The method to disrupt the olfactory epithelium via Triton X and ZnSO_4_ needs to be explicitly in the methods section, not merely a reference. One of the papers referenced, also neglected to put the method in the paper and only referenced another paper.

**Response:** Based on your suggestion, the detailed experimental procedure has been added to the Materials and methods (Lines 497-504).

5. Line 249. “absence of a decreased viral load”. It is unclear what they are trying to convey with this statement. Please clarify.

**Response:** Yes. The unclear wording has now been changed to “……probably associated with reduced inflammatory response in the nasal turbinate and no reduction in viral load” in Lines 371-372 in the revised manuscript.

6. The paper should reference Reyna, RA et al “Recovery of anosmia in hamsters infected with SARS-CoV-2 is correlated with repair of the olfactory epithelium” Scientific reports doi.org.10/1 038/S41598-021-04622-9. The findings should incorporated into the discussion.

**Response:** Thank you for your suggestion. We have read the paper carefully and incorporated their findings into the Discussion section of the revised manuscript (Lines 372-375).

7. The authors should look at a recent Trends in Neuroscience paper “The neuroinvasiveness, neurotropism and neurovirulence of SARS-CoV-2”. This may help as they revamp the discussion to talk about the hamster models and how these rodent systems relate to neuroinvasiveness of SARS-CoV-2. doi.org/10.1016/j.tins.20 22.02.006.

**Response:** Yes. We have read this reference carefully and updated the Discussion section of the revised manuscript.

**Reviewer #3:**

1. Revise English language! Especially the abstract and author summary need to be revised by a native speaker. Those two paragraphs are nearly impossible to understand due discrepancies in English language. It is getting better within the main text. However, I recommend a complete revision by a native English speaker

**Response:** Based on your suggestion, we have carefully revised the full text, and the manuscript has been proofread by a native English speaker. We hope that the revised manuscript has been substantially improved.

2. Throughout all figure captions be consistent in the description of your animal groups. Sometimes the age of mice is specified, sometimes not.

**Response:** We agree. We have added this information to the Figure legends section of the revised manuscript.

3. The authors are generally describing a set of very interesting and valuable data for this field of research! However, the lack of a general overview and clear experimental strategy is partially making it hard to follow.

**Response:** Based on your and other reviewers’ advice, a schematic experimental setup was added to the revised Fig.1A and a description of the study design was included in Lines 405-412 of the revised manuscript to allow the reader easily follow the workflow. In addition, we have further supplemented the details of the experimental strategies in the Methods and Figure legends sections.

**Responses to Specific comments:**

l.26: “biosafety betacoronavirus” is no scientifically correct term. It is a BSL-2 betacoronavirus

**Response:** Yes. As mentioned by you and other reviewers, we have deleted this term from the revised manuscript.

l.28: what is meant with “pathological test”? Please find a specific term. This leads to misunderstandings

**Response:** Yes. It has been changed to “histopathological examination” (Line 31).

l.34: I do not see the aspect of “low cost” being of such importance that it needs to be stated in the abstract. Money shouldn’t be a justification for the value and applicability of an animal model

**Response:** We agree. We have modified the Abstract and deleted “low cost” from the revised manuscript.

l.61: OE is susceptible to SARS-CoV-2 infection and not the other way around

**Response:** Yes. We have corrected the statement in the revised manuscript (Lines 364-365).

l.83: It is the order of Nidovirales (delete order in Nidoviralesorder)

**Response:** Yes. This taxonomic classification has now been corrected in the revised manuscript (Line 72).

ll.90-101: the whole paragraph describes your results. This is an extensive description of results for an introduction. Please keep it shorter and only give an overview of key findings.

**Response:** Yes. We have modified this description in the revised manuscript (Lines 109-117).

l.106: and throughout the whole manuscript: Don’t write 20µl of 10^4.66 TCID_50_/0.1ml but give a precise titer per animal! Since you know the volume and titer/volume and both are consistent for all animals, calculate the titer/animal.

**Response:** Yes. A precise titer per animal has been added throughout the whole manuscript.

l.107: Day of 100% mortality does vary between the two age groups. Should be mentioned here.

**Response:** Yes. We have clarified the statement in the revised manuscript (Lines 125-126).

ll.111: No differences between sexes but between ages. This finding should be mentioned here or above.

**Response:** Yes. The description has been added to the revised manuscript (Lines 130-132).

ll.112: In the figure caption it is described that only 6 week old mice were used for this test. Also for the description of the animal experiments, is there a reason to do some experiments with both age groups, some with 6 week old, some with 3 week old mice? Rational should be explained somewhere.

**Response:** In clinical practice, PHEV mainly affects suckling piglets under 4 weeks of age. Thus, we selected young mice at 3 and 6 weeks of age to mimic the clinical infection. For the behavioral experiments (revised Fig.2), we selected only 6-week-old mice for testing because these mice had fully developed organs and reached sexual maturity (PMID: 29988104; PMID: 33166988). Compared with 3-week-old mice, the behavioral evaluation of 6-week-old mice is more stable and reliable. Notably, previous studies have recommended using mice aged at least six weeks in protocols for behavioral experiments (PMID: 19575474; PMID: 33166988). For the remaining viral neuroinvasion test, we used immature 3-week-old mice because of the higher efficiency of PHEV invasion in younger mice.

Based on your comments, we have added additional explanations to the revised manuscript. Please see Lines 135-136 and 164-165 in the revised manuscript. In order to easy follow the workflow, a graphical experimental setup was added to the revised Fig.1A and a description of the study design was included in the Materials and methods section (Lines 405-412). In addition, we have carefully revised all of the figure legends and clearly stated the age of the mice employed.

l.132: Use another word than “propagated”. Which part of the brain?

**Response:** Yes. We have changed the statement to “PHEV preferentially infects the brain and spinal cord” in the revised manuscript (Line 159). As shown in the revised Fig 5A-F, viral RNA can be detected in the OE, OB, cerebrum, brainstem, cerebellum, and spinal cord at 5 dpi.

ll.140: Figure S2 is mentioned here, but not that it also shows that you observed no changes in peripheral tissues of infected mice. This observation with referring to Figure S2 should be included accordingly.

**Response:** Yes. This statement about Fig S2 (revised Fig S3) has been added to the appropriate places in the revised manuscript (Lines 161-163).

l.147: Again, why now 3 week old mice?

**Response:** Immature 3-week-old mice were used in the viral neuroinvasion test because intranasal inoculation of PHEV is more effective in young mice than adult mice (6-week-old) (PMID: 14693125).

l.151: Was that the nasal cavity also screened in this time course after infection (as described in table S1)? I think this table and the Figure 3A, B are highly interesting and can elucidate mechanisms of neuroinvasion. To have a complete picture, it would be perfect if the nasal turbinates would be included in the time course after infection.

**Response:** According to your suggestion, the olfactory epithelium in nasal turbinates of 1, 3 and 5 dpi have been included in the revised manuscript (revised Fig 4).

l.152: Use another word than “suffering”

**Response:** Yes. This term is now corrected in the revised manuscript (Line 190).

l.165: You observe reduced mortality but still the same neurological symptoms? Were functional assays (burying etc.) also performed with these groups?

**Response:** We do not agree. Our studies indicate that chemical treatment did not ultimately reduce mortality. Please see Lines 164-165 in the original manuscript and Lines 207-208 in the revised manuscript. According to previous publications, intranasal zinc administration has been reported as a cause of anosmia in humans and animals (PMID: 30202422; PMID: 16467707). Given the fact that functional assays (burying etc.) require a normal sense of smell, so we didn’t perform behavioral tests in the chemically treated mice.

l.166: You describe RNA data, which says nothing about infectious virus. Please re-phrase.

**Response:** Yes. The statement has been rephrased in the revised manuscript (Lines 212-214).

ll.222: This statement is not true. Syrian golden hamsters are currently the most widely used animal model for SARS-CoV-2 and also show neuroinvasion to a certain extent. Please also discuss the advantage of your model compared to the SARS-CoV-2 hamster model

**Response**：We agree. We have deleted this infelicitous statement and compared the advantages and disadvantages of the PHEV-infected BALB/c mouse model to the widely used Syrian golden hamster model in the Discussion section in the revised manuscript (Lines 363-375). In addition, a table comparing anosmia and brain infection among the PHEV BALB/c model, SARS-CoV-2-infected hamster model, SARS-CoV-2-infected humanized ACE2 mouse model and deceased COVID-19 patients has been added to the revised manuscript (S2 Table).

ll.227: To me this hypothesis mostly seems to be based on extrapolation. How can a model with another pathogen be used to study efficacy of therapeutic interventions? No direct comparions were performed that can proof the applicability of the model in this respect. Consider toning down the use of your model in this respect.

**Response:** Yes. We have modified the statement in the revised manuscript (Line 285-288).

ll.291: What is the specific use for this model in the future?

**Response:** Given that the high pathogenicity of human coronaviruses, relevant experiments must be conducted in a BSL-3 laboratory, which has undoubtedly hindered research progress. PHEV may serve as a potential prototype for elucidating the pathogenesis of coronavirus-associated neurological complications and olfactory and taste disorders under biosafety level 2 conditions.

ll.308: How were the polyclonal antibodies prepared? Either refer to previous publications or describe more details here. Were animals immunized? Which animals, which antigen, including adjuvants etc.?

**Response:** This information has been added to the Materials and methods section in the revised manuscript (Lines 437-443). Briefly, recombinant full-length PHEV-N protein was generated based on the sequence of the PHEV-CC14 strain (GenBank accession number: MF083115). The PHEV-N coding region was amplified by RT–PCR, and the PCR product was cloned into the pET-32a(+) vector (Novagen). The recombinant protein was expressed and purified by His-tag Purification Resin (P2233, Beyotime). Polyclonal antibody was prepared in New Zealand white rabbits. For primary immunizations, 100 μg antigen was mixed with an equal volume of Freund’s incomplete adjuvant and injected subcutaneously at eight different locations. Rabbits were boosted three times at 2-week intervals. The immune response was monitored using IFA and ELISA.

ll.318: Please be more precise about the use of animals and different sub-experiments. It is very confusing to get an overview which and how many animals were used to answer the different questions, addressed by different experiments. See also general comment

**Response:** We agree. In order to easy follow the workflow, a graphical experimental setup was added to the revised Fig.1A and a description of the study design was included in the Materials and methods section (Lines 405-412). In addition, we have carefully revised all of the figure legends and clearly stated the age and number of mice employed.

l.634: Please change “viral loads” to “viral genome loads”

**Response:** Yes. This information has been modified in the revised manuscript (Line 930).

l.635: Why is it n=4 for titrations and n=6 for PCR?

**Response:** We apologize for this mistake. In this experiment, n=6 mice for both qRT–PCR analysis and viral titration. This information has been corrected in the revised manuscript (Lines 932 and Fig 3B).

l.644: Sacrificed at what day post infection?

**Response:** The infected mice were sacrificed at 1, 3 and 5 dpi, respectively. We have added this information in the revised manuscript (Lines 941-942).

l.654: What is the added value of the PBS group compared to the untreated animals? Why do all groups have different group sizes?

**Response:** ZnSO_4_ and Triton X-100 were dissolved in PBS, and we used the PBS+PHEV group as a positive control. Mice in the untreated group did not receive any treatment as a negative control. Considering that ZnSO_4_

and Triton X-100 treatments may cause the death of mice, the number of mice in the chemical treatment group was larger than that in the other two groups. For brevity and clarity, we have added the number of mice in every group to the revised Fig 6A.

l.656: Which of the above mentioned groups is subset (B) describing? And How can it be 6 mice per time point (3 in total) if one treatment group consists of maximum 16 animals?

**Response:** The data shown in the original Figure 4B (revised Fig 6B) were obtained from an experiment independent of the data shown in the original Figure 4A (revised Fig 6A). The mice in Figure 4B (revised Fig 6B) were sacrificed at 4, 5, and 7 dpi, while the mice in the original (revised Fig 6A) died spontaneously. For a clearer description of the experiment, we revised the figure legend in the revised manuscript (Lines 960-965).

ll.657: Which animals are described in (C) and (D)?

**Response:** The original Fig 4C and 4D are reorganized as Fig 7A and 7B, and the figure legend has been updated in the revised manuscript (Lines 966-973).

l.691: n=7 is described here, whereas in caption of Figure 1 it is n=5. Or are these separate experiments?

**Response:** We apologize for this typo in our text, and we have corrected this mistake in the revised manuscript (Line 1011).

Figure 1 D-L: I am missing the mock-controls or are they reflected in the 0 dpi data? Don’t you assume a behavioral adaptation of animals over time and wouldn’t it be ideal to include measurements for mock-controls at all time points?

**Response:** The M in the x-coordinate of revised Fig 2A-2C represents the mock controls, and we have added this information to the legend of the revised manuscript (Line 915). Before this experiment, mice were continuously exposed to both 1% sucrose water and regular water for 48 h to adaptation (PMID: 29988104). The same group of mice was tested on different days to eliminate individual differences between different mice. Therefore, we did not include measurements for mock controls at all time points

Figure 2 A, B: Use a Log10 y-axis for both graphs

**Response:** Yes. The axes have been corrected in the revised Fig.3A-B.

Figure 3 A, B: Very impressive images. Was this also done for the 1 dpi time point and for the nasal cavity?

**Response:** Yes. The olfactory epithelium of the nasal cavity and brains at 1, 3 and 5 dpi have been included in the revised manuscript (revised Fig 4).
