# Supplementary material for: PHEV infection: A promising model of betacoronavirus-associated neurological and olfactory dysfunction
Source: PLoS Pathog. 2022 Jun 27;18(6):e1010667. doi: 10.1371/journal.ppat.1010667 (PMC9282652; doi:10.1371/journal.ppat.1010667)
Supplement: Attachment — Submitted filename: Response letter-R2-PLOS Pathogens-20220605.docx [file ppat.1010667.s009.docx]

**Response letter**

Dear Editors,

Thank you very much for your kind letter. We have carefully revised the manuscript in accordance with the editors’ and reviewers’ comments. All concerns and questions have been addressed point-by-point below.

We hope our manuscript is now suitable for publication in *PLOS Pathogens*.

Kind regards,

Wenqi He

Professor of College of Veterinary Medicine, Jilin University, China

On behalf of all authors.

June 5, 2022

Responses to reviewers’ comments

1. In the authors summary the focus is the usefulness of PHEV infections in mice as a model to study the pathogenesis neurological complications of SARS-CoV-2. Please modify this according to the previous comments of the reviewers.

**Response:** Thank you for your insightful comments. We have considered your advice and carefully modified the statement in the Author summary section. We have re-defined the meaning of anosmia and made the summary focus on the unraveling of viral pathogenesis. (Lines 43-51)

2. The discussion is too long and too much focused on SARS-CoV-2. In addition, it lacks a thorough discussion on the limitations of experimental PHEV infection in mice as a model for SARS-CoV-2 associated CNS complications. It is for example not included that there is a large difference in the ability of SARS-CoV-2 and PHEV to spread throughout the CNS. Although SARS-CoV-2 virus antigen/RNA is occasionally detected in the CNS of humans (or relevant animal models), it does NOT replicate/spread efficiently throughout the CNS causing a lethal encephalitis. This is an crucial difference, and for this reason the mechanism of SARS-CoV-2 associated neurological disease (including anosmia and ageusia) are likely very different.

**Response:** Thank you for your suggestions. We have modified the statement in the Discussion section of the revised manuscript. (Lines 276-344)

(1) Based on your comments, we have removed the dispensable statements on SARS-CoV-2 and focused on what our findings mean in the context of PHEV infection in order to more concise and tightly argued.
(2) In addition, we have added a thorough discussion on the limitations of experimental PHEV infection in mice as a model for SARS-CoV-2 associated CNS complications in the revised manuscript (Lines 325-344). We hope the revised manuscript has been substantially improved.
